# Recently Emerged Novel Henipa-like Viruses: Shining a Spotlight on the Shrew

**DOI:** 10.3390/v15122407

**Published:** 2023-12-11

**Authors:** Sarah Caruso, Sarah J. Edwards

**Affiliations:** Australian Centre for Disease Preparedness, Health & Biosecurity, Commonwealth Scientific and Industrial Research Organisation (CSIRO), 5 Portarlington Road, East Geelong, VIC 3219, Australia; sarah.caruso@csiro.au

**Keywords:** henipavirus, paramyxovirus, pteropid bat, shrew, zoonotic, virus, outbreak potential, reservoir host

## Abstract

Henipaviruses are zoonotic viruses, including some highly pathogenic and capable of serious disease and high fatality rates in both animals and humans. Hendra virus and Nipah virus are the most notable henipaviruses, resulting in significant outbreaks across South Asia, South-East Asia, and Australia. Pteropid fruit bats have been identified as key zoonotic reservoirs; however, the increased discovery of henipaviruses outside the geographic distribution of Pteropid fruit bats and the detection of novel henipa-like viruses in other species such as the shrew, rat, and opossum suggest that Pteropid bats are not the sole reservoir for henipaviruses. In this review, we provide an update on henipavirus spillover events and describe the recent detection of novel unclassified henipaviruses, with a strong focus on the shrew and its emerging role as a key host of henipaviruses.

## 1. Introduction

According to the International Committee on Taxonomy of Viruses (ICTV), the genus *Henipavirus* comprises five species: Cedar virus (CedV), Ghanian bat virus (GhV; formerly Kumasi virus), Mòjiāng virus (MojV), Hendra virus (HeV), and Nipah virus (NiV) [1]. Henipaviruses, belonging to the family *Paramyxoviridae*, are enveloped, negative-sense RNA viruses, with prototypic members HeV and NiV classified as risk group 4 pathogens [2]. Infection with HeV and NiV has been associated with severe respiratory illness, neurological disease, febrile illness, and high fatality in animals and humans [3,4,5,6,7,8]. With an ability to infect a range of species, often resulting in fatality, coupled with a lack of human vaccines and therapeutics, henipaviruses remain an ongoing critical threat to both animals and human public health.

## 2. Emergence of Henipaviruses

Henipaviruses were first detected in 1994 in Brisbane, Australia, following the outbreak of a novel etiological agent in horses and humans. HeV was initially identified as Equine morbillivirus and resulted in the death of 20 horses and a horse trainer following close contact with an infected horse [9,10,11]. Since the emergence of HeV, there have been 89 confirmed cases in horses and 7 human cases, of which 4 people have died [12,13,14]. In 2013, surveillance of Australian flying foxes following an extreme heat mortality event resulted in the detection and identification of a novel HeV genotype [15]. Designated HeV-g2, the full-length HeV variant genome shows an 83.6% nucleotide identity to HeV prototype strain (GenBank accession no: AF017149)—the largest variation observed amongst HeV isolates which previously saw <1% variation [15,16].

Emerging in Malaysia less than five years after the initial HeV outbreak, NiV infection caused 105 human fatalities out of 265 confirmed cases [17], and the spillover into humans was associated with respiratory illness in pigs. Infection with NiV caused fatal neurological disease and respiratory illness in pig farmers, with subsequent outbreaks across Malaysia and Singapore—the latter, in abattoir workers, a result of slaughtering infected pigs imported from Malaysia [17,18,19]. Following the identification of pigs as a host reservoir (both in farming and abattoirs), over one million pigs were culled in an effort to contain the outbreak and prevent future spread. This effort has been successful and resulted in no further NiV outbreaks in Malaysia or Singapore [17].

In 2001 and 2003, outbreaks of NiV in the South Asian region of Bangladesh were reported following human cases of febrile neurological disease and subsequent fatalities [4,20]. Serological testing for NiV-specific antibodies confirmed NiV as the causative agent [4]. Since its first emergence in Bangladesh, NiV outbreaks have occurred almost annually within the India–Bangladesh region, demonstrating high fatality rates in humans (Table 1). Unlike the NiV Malaysia (NiV_Mal_) outbreak, the NiV Bangladesh strain has demonstrated person-to-person transmission [21]. More recently, an outbreak of severe disease in the southern Philippines [22] included symptoms of influenza-like illness, meningitis, and encephalitis, with many cases resulting in death in horses and humans. Serological analysis and a 71-base-pair sequence read aligning the NiV phosphoprotein gene suggest the causative agent to be NiV, or a NiV-like virus.

A third henipavirus was isolated from Pteropid bat urine collected in Queensland, Australia, in 2012 [31]. Having similar genome size and organisation as HeV and NiV, CedV showed cross-reactivity with henipavirus antigens and was found to also utilise ephrin receptors for infection; however, CedV did not cause clinical disease during experimental challenge of ferrets and guinea pigs. The lack of RNA editing of the CedV P gene is thought to contribute to this non-pathogenic outcome during infection of animal species normally susceptible to henipaviruses. CedV has also been isolated from Grey-headed flying foxes in Victoria, Australia, at a location over 1800 km away from the isolation of the CedV prototype strain [32].

Two additional viruses have been added to the henipavirus genus based on sequence alignment; however, viral isolates have not been obtained. Detected in pooled faeces belonging to African Straw-coloured fruit bats located in Kumasi, Ghana, the henipavirus GhV shows sequence homology to the L gene of NiV_Mal_ [33,34]. MojV, detected in an abandoned mine in Mòjiāng Hani Autonomous County, Yunnan Province, China, was identified by PCR of rectal swabs taken from cave rats (*Rattus flavipectus*) at a site where three patients had previously died of severe pneumonia complications after working in the mine in 2012 [35]. Although the mine was identified as the likely site of the outbreak, the direct link that MojV was the etiological agent that caused the deaths remains unconfirmed.

Since the identification of the five henipaviruses we have described here, multiple novel unclassified henipa-like genomes have been detected and/or isolated in locations beyond the currently known geological range, and a broadening of the host species diversity has been observed.

## 3. The Unclassified Henipa-like Viruses

Having genome size and organisation typical of henipaviruses, Gamak virus (GAKV) and Daeryong virus (DARV) were detected during small animal surveillance in the Republic of Korea between 2017 and 2018 [36] using next-generation sequencing (NGS), with GAVK subsequently isolated from kidney tissue homogenate [36]. Interestingly, GAKV and DARV were identified in the mammalian shrew (*Crocidura lasiura* and *Crocidura shantungensis*, respectively), a genus that was new to the henipavirus landscape. These findings broadened the diversity of known permissive hosts and their potential role in henipavirus spillover events into the human population.

**Figure 1:**
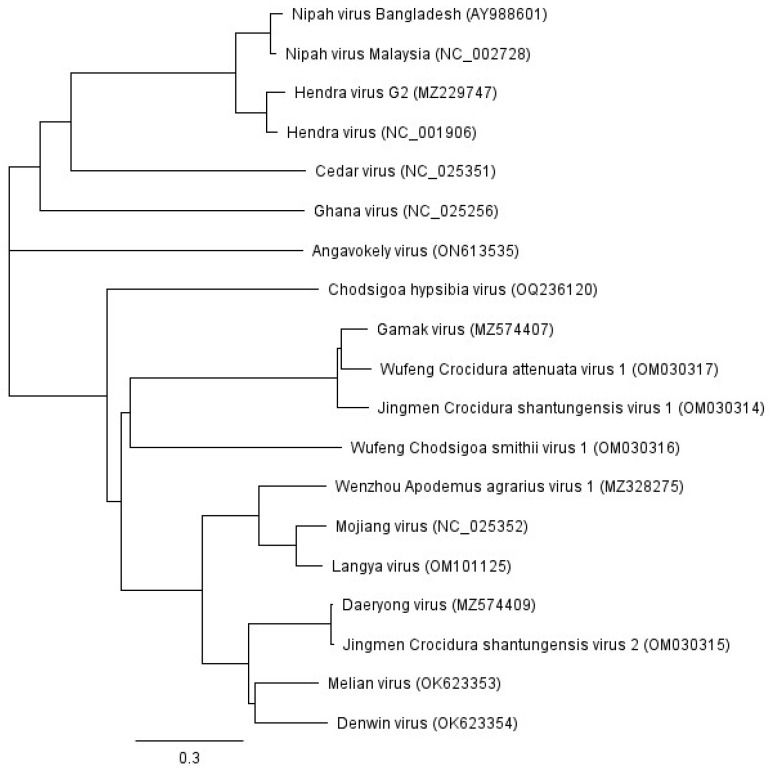
Phylogenetic analysis of henipavirus attachment glycoprotein sequences (classified and unclassified). GenBank accession numbers are provided in parentheses.

Identified in 2019, Langya virus (LayV) was detected and isolated from a throat swab sample taken from a febrile patient in the Shandong and Henan provinces of China [37]. Found during sentinel surveillance of patients exhibiting acute fever and recent animal exposure history, LayV was detected in 35 infected patients. It is important to note that LayV infection within this cohort was not fatal, and in comparison to the clinical manifestation of HeV and NiV infection, a milder disease of febrile illness was observed. Additionally, patient samples were collected between 2018 and 2022, indicating that spillover events may have been steadily occurring during this period. Metagenomic analysis revealed that the 18.4 kilobase genome was similar in length and organisation to other henipaviruses, while phylogenetic analysis of the attachment glycoprotein (G) amino acid sequence shows a close relatedness to MojV (Figure 1). An investigation to identify the animal(s) responsible for the zoonotic event involved the screening of small wild animals (25 species in total). LayV-specific RNA was detected in shrews (71 out of 262 (27%)), specifically *C. lasiura* and *C. shantungensis*—the same shrew species harbouring GAKV and DARV, respectively. The detection and prevalence of LayV in shrews suggests that shrews are a natural reservoir of LayV.

Angavokely virus (AngV) was identified from a urine sample obtained from a Malagasy fruit bat (*Eidolon dupreanum*) on the island of Madagascar in 2019 [38]. Although the virus was not isolated, the sample produced sufficient reads for the assembly of the complete coding sequence; however, whole-genome assembly was not possible (the recovered genome lacks part of the 5′ untranslated region of the nucleocapsid open reading frame). Interestingly, phylogenetic analysis of the RNA-dependent-RNA polymerase (L) protein amino acid sequence shows that AngV clusters separately from the other bat-borne henipaviruses (Figure 1)—a divergence that occurred approximately 9800 years ago [38].

Separate from the bat-borne henipaviruses, melian virus (MeliV) identified in *Crocidura grandiceps* in the Nzérékoré Region of Guinea, and denwin virus (DewV), identified in *Crocidura russula* in Belgium, Brussels [39], have further emphasised the important role shrews play as host reservoirs of henipaviruses. Although the identification of these viruses did not result in virus isolates, whole-genome sequences were determined at 19.9 kb and 19.7 kb for MeliV and DewV, respectively [39]. Interestingly, MeliV and DewV were obtained from different continents; however, phylogenetic analysis shows that they cluster closely together (Figure 1) and are most closely related to the shrew-borne henipavirus DARV. It is also important to note that both MeliV and DewV encode an additional open reading frame (X) of unknown function situated between the matrix and fusion genes [39].

Most recently published, a partial sequence of Peixe-Boi virus (PBV) was detected in Brazilian opossums (*Marmosa demerarae*) during the surveillance of small wild animals in Pará State, Brazil, in 2015 [40]. Partial sequence was obtained from tissue samples resulting in a 2377-nucleotide sequence aligning to the L gene of NiV Malaysia prototype strain. Phylogenetic analysis on the partial sequence suggests that PBV clusters independently from the rodent/shrew-associated and bat-borne subclades. Although only a partial sequence, the detection of PBV is of great interest, being the first sequence confirmation of henipa-like viruses in the Americas.

The complete genomes of additional henipa-like viruses have recently been submitted to GenBank (Table 2); however, further details regarding these viruses have not been published. This includes Wenzhou Apodemus agrarius henipavirus 1 (GenBank: MZ328275.1), detected in the striped field mouse (*Apodemus agrarius*) in China, and the following which were discovered in shrews: Wufeng Chodsigoa smithii henipavirus 1 (GenBank: OM030316.1), detected in *Chodsigoa smithii* shrews in China; Wufeng Crocidura attenuata henipavirus 1 (GenBank: OM030317.1), detected in *Crocidura attenuate* shrews in China; and Jingmen Crocidura shantungensis henipavirus 1 (GenBank: OM030314.1) and Jingmen Crocidura shantungensis henipavirus 2 (GenBank: OM030315.1), both detected in *C. shantungensis* shrews in China. Numerous partial sequences of novel henipa-like viruses have also been submitted to GenBank.

**Figure 2:**
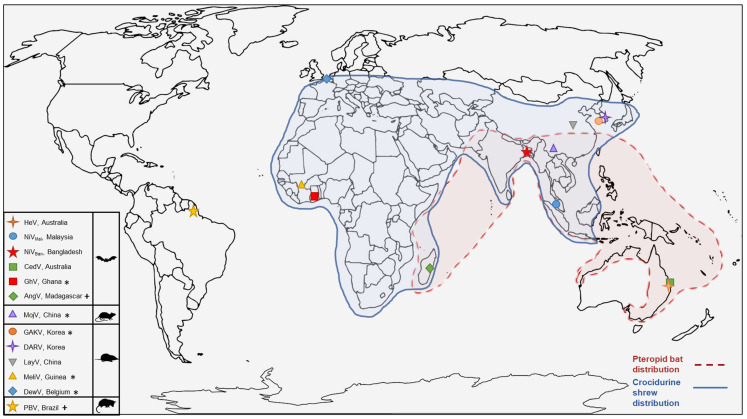
Locations where henipa- and henipa-like viruses were originally detected and the distribution range of two reservoir hosts: the Pteropid fruit bat and crocidurine shrew. The animal host of detection/isolation is shown in the legend. ‘*’ denotes where whole genome was detected in the absence of a viral isolate. ‘+’ denotes that a partial sequence was obtained. Global distribution range for the Pteropid fruit bat taken from [5,41]. Crocidurine shrew distribution range taken from [42].

The pathogenicity of many recently identified novel henipa-like viruses is unknown, and therefore, their zoonotic potential resulting in fatal outcomes is also not known. Given the wide geographical distribution of the henipaviruses and henipa-like viruses across multiple continents (Figure 2), the detection of these viruses is likely to continue, and the potential for further spillover events occurring is probable.

## 4. Natural Reservoirs for Henipaviruses

The natural reservoir of HeV, NiV, and CedV is the Pteropid bat, belonging to the order *Chiroptera* [31,43,44,45]. In their natural habitat, pteropid fruit bats are commonly found within the forest landscape roosting in high-density populations amongst trees; however, their roosting sites frequently coincide with the location of human residential areas and farms—an interaction commonly influenced by human activities [46]. HeV spillover events have been linked to the behavioural changes of bats in response to environmental factors including change in land use, climate change, habitat loss, and food shortages [47,48,49]. The link between people living in areas subjected to deforestation in Cameroon and the prevalence of NiV seropositive individuals further highlights the role bats play as reservoir hosts during zoonotic spillover events [50]. Transmission of HeV and NiV_Mal_ has been attributed to bats roosting close to farms holding horses and pigs, respectively [44,45], with those animals acting as amplifying vectors for human infection. In Bangladesh, the unique transmission of NiV directly from bats to humans is apparent through the human consumption of raw date palm sap previously contaminated via bat saliva and urine [51,52,53,54]. The most likely route of transmission from bats to subsequent hosts is through virus shedding in urine with henipaviruses frequently detected and/or isolated from bat urine [31,38,45,48,55,56,57], including a study recognising a parallel between the increased detection of HeV from pooled bat urine and the increase in spillover events within the same time frame [56]. Evidence of henipa-like viruses has been detected in blood, rectal, and nasal samples of bats; however, it is unknown if these sample types contribute to transmission [48]. In addition to bodily excretions, NiV has been detected in partially eaten fruit left behind by bats, demonstrating a possible intermediate environmental source for virus spillover [45].

While early outbreaks of henipaviruses have correlated to geographic regions where Pteropid bats are found, many new discoveries expand beyond these boundaries (Figure 2) [58]. The detection of novel henipa-like viruses in Africa, Europe, and South America no longer falls within the natural distribution range of Pteropid bats, emphasising the need to explore other potential host reservoirs. In Ghana, bats still appear to be a key zoonotic reservoir with GhV sequences detected from *Eidolon* bat species indicating additional bat species as reservoirs for henipaviruses [33]. Furthermore, there is serological evidence of henipa-like viruses circulating in bat populations other than *Pteropus*, including Trinidad, China, Madagascar, Ghana, and Cameroon [50,59,60,61,62].

It is important to note that like the identification of MojV in cave rats, many of the recent henipa-like virus discoveries include sequences and/or virus isolates from small mammals including shrews, rats, and opossums [35,36,37,39,40]. To date, eight full-genome novel henipa-like viruses have been associated with shrews and one other partial henipavirus-like sequence in opossums (Table 2), and until the emergence of LayV in 2019, henipavirus-associated outbreaks were only attributed to zoonotic transmission from Pteropid fruit bats. The detection of henipaviruses in rats and shrews suggests that henipaviruses could spillover from other species, and thus, predicting future henipavirus outbreaks would prove difficult. Furthermore, the shrew-isolated virus GAKV has shown to infect human lung epithelial cells in vitro [36]; therefore, the risk of zoonotic transmission and subsequent human infection is possible.

## 5. Shrews: A Henipavirus Reservoir Host

Shrews are ancient, small mouse-like terrestrial mammals that can be found throughout all regions of the globe except Australia, New Zealand, New Guinea, and Antarctica [63,64]. Shrews belong to the superorder of placental mammals *Laurasiatheria*, order *Eulipotyphla*, family *Soricidae* [65], and are further divided into three subfamilies: *Soricinae*, *Crocidurinae*, and *Myosoricinae*. Interestingly, the relatedness of the shrew and bat is believed to have stemmed from a common ancestor almost 100 million years ago, when the order of *Eulipotyphla* (a remnant of the previous classification order *Insectivora*) is believed to have diverged from its last Chiropteran common ancestor [66]. The same splitting event may have also led to the creation of the lineage to which the rodents now belong (order *Rodentia*) [66]. Fossil evidence has suggested that shrews belonging to the family *Soricidae* first evolved between 30 to 40 million years ago [67], placing these ancient mammals on the planet shortly after the dinosaurs disappeared.

Comprising over 370 different species, the *Soricidae* family is one of the largest and most abundant of the mammals, with a diverse and complex taxonomy [63], and as such, many characteristics of shrews are varied between species. Shrews can be found in diverse ecological niches ranging from arid deserts and tropical rainforests to semiaquatic environments [64], and therefore, their size and physical attributes vary accordingly. Shrews such as the Etruscan shrew (*Suncus etruscus*) are smaller in size than a mouse and weigh less than 2 g—the smallest terrestrial mammal recorded [68]. In contrast, the large musk shrew (*Suncus murinus*) is the largest recorded species of shrew, weighing up to 175 g [42,69]. Shrews are commonly predatorial foragers and largely insectivorous; however, depending on species, the diet of the shrew can also consist of plants, earthworms, molluscs such as snails, crabs, fish, and other small vertebrates [64]. Given their small body size, shrews have very high energy requirements, and in comparison to other small mammals of similar size, shrews have high energy costs of reproduction [70].

Shrews are typically solitary creatures and remain asocial except for mating requirements [42]; however, there are some shrews that are an exception. Depending on the species, *Soricidae* shrews can be territorial or tolerant and social with other shrews [71], which may influence the circulation of pathogens between individuals and within populations. For example, the presence of male shrews in the nest is not normally tolerated by females, particularly when young are present; however, males of the genus *C. russula* (of which DewV was detected) are present in the nest with both the female and young [42]. Furthermore, the territorial range of *C. russula* is quite small (75–395 m^2^), and there is substantial overlap between individual territories [42], providing opportunity for the intraspecies spread of pathogens.

## 6. Shrews as Reservoir Hosts for Viruses

The zoonotic reservoir potential of shrews is not a new concept, and shrews have been identified as hosts of other zoonotic viruses including bornaviruses [72], flaviviruses [73], and hantaviruses [74,75,76]. Given the prevalence of zoonotic and potentially zoonotic viruses found in shrews, geographical areas where shrews are endemic are often considered when screening small mammals for novel viruses [39,77,78]. As a result, several novel viruses have been identified during surveillance efforts in shrews. Recently detected in high frequency, during surveillance of Asian house shrews in Singapore, Cencurut virus (CENV) is a novel orthonairovirus belonging to the family *Nairoviridae* of viruses with known potential for zoonotic spillover [79]. The high CENV-infection frequency in shrews coupled with high human density living in many parts of Asia may suggest a higher probability for zoonotic spillover events to occur in the future.

Shrews are endemic throughout the European continent, and as such, many shrew-borne viruses have been identified. PCR analysis of brain tissues collected from *Crocidura leucodon* shrews in Switzerland detected the presence of Borna disease virus during sampling activities searching for reservoir hosts and vectors specific to bornaviruses [72]. Shrews were also implicated as the likely host reservoir of the flavivirus, Powassan (POW) virus lineage 2 (or deer tick virus; DTV) in North America [73]. DTV is a tick-borne RNA virus and is known to cause acute encephalitic disease in humans, with an increase in the prevalence of human cases over the last two decades [80]. Of the 20 DTV-infected ticks that were collected in the Northeastern United States, 65% of ticks were shown to have previously obtained a blood meal from a shrew [73]. Thus, although DTV is a tick-borne virus, there is opportunity for shrews to act as an amplifying host arising from cross-species transmission, increasing the occurrence of infection events between ticks and humans.

There are 109 recognised species of Crociduran shrew found in Africa [81], and as such, the detection of viruses in the shrew population of Africa is common. For example, Hepatitis B viruses (HBV; genus *Orthohepadnavirus*) have been detected in African shrews, and although the prevalence of HBV was low in the 693 shrews screened, sampling was conducted over a broad geographic range, suggesting that these viruses are widespread and are persistently maintained at low levels within the shrew population [82]. HBV has a devastating effect on human health and is responsible for approximately 887,000 deaths worldwide [83], and it is not certain if spillover events from shrews contribute to human HBV cases; however, the wide distribution of shrews in areas of human populations suggests that it is possible.

Hantaviruses (genus: *Hantavirus*, family: *Bunyaviridae*) have the ability to cause serious human disease, including haemorrhagic fever with renal syndrome (HFRS) and hantavirus pulmonary syndrome (HPS), and are therefore a high priority in zoonotic research [74]. Although initially thought to be primarily rodent-borne viruses, hantaviruses are commonly detected in shrews, and many novel shrew-borne hantaviruses have been identified during surveillance and screening [74,75,76,84]. The first hantavirus to be associated with shrews was Thottapalayam virus (TPMV), isolated from *Suncus murinus* musk shrews in India in 1971 [85]. Since the discovery of TPMV, many novel hantaviruses have been found in shrews across Asia, Africa, and Europe. The isolation and/or detection of hantaviruses has occurred in geographically distant regions of the globe. These include Imjin virus (MJNV), isolated from the lung of *C. lasiura* shrews in the Republic of Korea [74]; Azagny virus (AZGV), detected in *Crocidura obscurior* shrews in Côte d’Ivoire [76]; and Seewis hantavirus (SWSV), detected in *Sorex* shrews from Sweden and central Europe including Switzerland, Germany, the Czech Republic, and Slovakia [86,87,88]. Similar to henipavirus transmission, hantaviruses are transmissible to humans via inhalation of respiratory secretions [87,89], highlighting a key potential transmission mode that could see shrew-borne henipaviruses spillover into the human population.

Generally, the sampling of small mammals for the purpose of novel virus discovery traps more rodent species, with shrews representing only a small number of mammals screened in such studies, yet the frequency of shrew-associated virus discovery suggests a prevalence of shrew viral reservoirs. It is therefore important to consider the ecology and behaviours of shrews in relation to possible infectious zoonotic diseases. DNA analysis of digested prey species in the faeces of the leopard cat (*Prionailurus bengalensis*) in continental Asia has identified the remains of two shrew species, *C. lasiura* and *C. shantungensis* [90]. Interestingly, these are the shrew species associated with henipa-like viruses GAKV and DARV [36] and Jingmen Crocidura shantungensis henipavirus 1 and Jingmen Crocidura shantungensis henipavirus 2. Catching and consumption of virus-infected shrews by higher predatory animals may create an opportunity for cross-species transmission events, and subsequently, these predatory species may have a potential to function as amplifying hosts contributing to the persistence and spread of these viruses.

### 6.1. Interactions between Shrews and Humans

Shrews are an invasive species to many geographical locations, rapidly adapting and becoming established in new environments, which when acting as a zoonotic reservoir has serious implications in the spread of disease [91,92,93]. Shrews can be found in both rural and urban areas, relying on shelter, warmth, and food during cooler weather, placing them in close proximity to livestock, agriculture, and areas of human populations [92,94,95,96]. The roosting of bats in and around agricultural areas has been shown to correlate to an increased risk of spillover of HeV and NiV [47,51,97,98]; therefore, it is plausible that shrews may pose a similar risk given their wide distribution. The introduction of shrews to new geographical locations has been facilitated in a number of ways including the formation and erosion of geological land bridges and the unintentional introduction of shrews via human influence such as the movement of shipping vessels [99,100].

A recent study conducted in both Portugal and Poland observed the behavioural characteristics of two species of shrew, *Sorex araneus* and *C. russula*, found in contrasting environments of urban and rural settings [96]. It was found that shrews living within urban habitats were more likely to display bolder behaviours. This characteristic was increasingly noticeable in the more synurbanised *C. russula*, allowing the species to better adapt and overcome the many challenges and changes animals must endure when living in urban environments [96]. One could theorise that the bolder behaviour of these shrews may encourage a greater frequency of contact between shrews and humans, and that could in turn increase the potential for zoonotic spillover events of pathogenic viruses. Further understanding of the transmission of shrew-borne henipaviruses will provide more information on how habitat and behaviour may contribute to the potential of future outbreaks.

### 6.2. Novel Henipaviruses and Shrews

In a study published in 2014, the screening of wild rodents and shrews in Zambia provided the first indication that shrews may be harbouring paramyxoviruses [78]. From wild shrews trapped for this study, RT-PCR analysis of kidney tissues detected henipa-like paramyxovirus sequence in 12 of the 31 shrews tested. To date, we have seen the isolation of two novel henipaviruses from shrews and the detection of an additional seven novel henipavirus genomes (see Table 2). The detection of henipa-like viruses in shrews has largely been within the genus *Crocidura*, commonly known as white-toothed shrews, or musk shrews. These shrews are found abundantly across Europe, Asia, and Africa [101] (Figure 2), and accordingly, henipa-like viruses from crocidurine shrews have been identified in Korea, Guinea, Belgium, China, and Zambia [36,37,39,78]. Henipa-like viruses *Chodsigoa hypsibia henipavirus* (GenBank OQ236120.1) and *Wufeng Chodsigoa smithii henipavirus 1* (GenBank OM30316.1) have also been detected within the Chodsigoa genus of shrews.

While LayV was initially observed causing disease in humans, the subsequent serosurvey and detection of LayV-induced antibodies within domestic and small wild animals has highlighted the potential for shrew-borne spillover into humans [37]. Henipa-like viruses GAKV and DARV were the only two henipaviruses discovered in shrews during a study that identified a number of novel paramyxoviruses in small mammals in Korea [39], which also highlights the key role shrews may play in the persistence and recurring emergence of these viruses.

Similarly, MeliV and DewV were detected in crocidurine shrews from Guinea and Belgium, respectively [36]. Phylogenetic analysis shows that these henipaviruses cluster closer to the rat-derived henipavirus MojV (Figure 1) than to the previously described bat-borne henipaviruses [36,39], thus resulting in a distinct lineage for shrew-borne henipaviruses [39]. The observation of virus relatedness has been observed previously when other shrew-borne paramyxoviruses were confirmed as phylogenetically distinct from their rodent-derived or bat-derived counterparts [74,76,77,86,102]. For example, this phylogenetic difference may be contributing to the inability of MJNV to cross-neutralise with rodent-derived hantaviruses and, conversely, display some cross-neutralisation with other shrew-derived hantaviruses such as TPMV [74]. Interestingly, PBV, detected in Brazilian opossums, was distinct from both bat-derived and shrew-derived henipaviruses [40]; however, this analysis is based on the alignment of a 2377 nt fragment, and no other genomic sequence was obtained.

As we have described, the predominant route of henipavirus transmission is via excretions from primarily bat urine. The number of human cases of LayV infection throughout 2018–2022 indicates a sustained spillover during the three-year period [37], and therefore, investigating mechanisms of zoonotic transmission from newly identified animal hosts is of significant importance. Several viruses harboured by shrews have been investigated in this context. Viral shedding of infectious Borna Disease virus-1 (BVD-1) has been detected in the saliva, urine, and faeces of shrews [103], whilst the tick-mediated transmission of the flavivirus, POW virus, has shown that infected ticks are likely to feed on shrews resulting in virus transmission [73]. So far, all shrew-derived henipa-like viruses have been identified in tissues and serum [36,37,39], and it is unclear if shrews shed these viruses via urine, faeces, or saliva. Given that some species of shrew habitually live in close proximity to human dwellings, a greater understanding of shrew-mediated transmission of these viruses is warranted.

There is limited evidence of viral transmission from shrews to horses, possibly, in part, as shrews are not endemic to Australia—where HeV spilled over into horses. A study aiming to identify the natural reservoir of BDV found that the BDV genome sequence obtained from shrews shared sequence homology with a virus that was detected from a nearby horse fatality [72]. This not only implicates shrews as a potential host reservoir but also highlights the proximity of shrews harbouring such viruses to horses and other livestock [72]. High virus titres of BDV have been shown to be present in the urine of infected rats [104], and it is possible that shrews may harbour these viruses in a similar magnitude. The possibility of shrews harbouring henipaviruses in a similar fashion is of great interest, as are the implications of cross-species transmission into livestock.

## 7. Summary

The increase in henipa-like viruses identified over the last decade, and their broadened global distribution, strongly suggests that Pteropid bats are not the sole animal reservoir, with strong evidence implicating shrews, mice, rats, and opossums. We currently do not know the pathogenicity and spillover risks of many of these viruses, nor do we know their potential for disease and mortalities in humans or the threat to livestock industries. As new potential animal reservoirs are identified, surveillance and characterisation of the new vector species, such as the shrew, should be prioritised, as it is possible that alternate routes of zoonotic transmission exist. As novel henipaviruses continue to emerge in new regions, the characterisation of emerging henipa-like viruses with a strong focus on virus pathogenicity, differences between reservoir hosts, and intraspecies transmission will be critical for predicting and preparing for potential future zoonotic events. The devastation caused during the HeV and NiV outbreaks is a reminder that the emergence of henipa-like viruses can result in deadly consequences and that understanding these viruses and their reservoir hosts is important for preparedness for future spillover events.

## Figures and Tables

**Table 1:** Global recorded human cases of NiV and NiV-like outbreaks by year and country as of November 2023.

Year (s)	Country	Confirmed Cases	Deaths	% Case Fatality	Reference
1998–1999	Malaysia	265	105	40	[19]
1999	Singapore	11	1	9	[18]
2001	India	66	45	68	[23]
2001	Bangladesh	13	9	69	[24]
2003		12	8	67	
2004		67	50	75	
2005		13	11	85	
2007		18	9	50	
2007	India	5	5	100	[25]
2008	Bangladesh	11	9	82	[24]
2009		4	0	0	
2010		18	16	89	
2011		42	36	86	
2012		18	13	72	
2013		26	22	85	
2014		38	15	39	
2014	Philippines	17	9	53	[22]
2015	Bangladesh	18	11	61	[24]
2017		3	2	67	
2018	India	18	16	89	[26]
2018	Bangladesh	4	3	75	[24]
2019		8	7	88	
2019	India	1	0	0	[27]
2020	Bangladesh	6	4	67	[24]
2021	India	1	1	100	[28]
2021	Bangladesh	2	0	0	[24]
2022		3	2	67	[29]
2023		13	8	73	
2023	India	6	2	34	[30]
Malaysia, Singapore, and Philippines		293	115	34	
India		95	69	73	
Bangladesh		335	235	65	
**Total**		**723**	**412**	**58**	

**Table 2:** Henipa- and henipa-like viruses and their proposed reservoir. Viruses listed are where full-length genome and/or an isolate was obtained, with the exception of AngV and PBV, included here to highlight their unique geographic locations.

Henipa/Henipa-like Virus	Primary Site of Detection	Isolate (I) or Sequence Only (S)	Proposed Reservoir	Reference (or GenBank Accession Number)
Hendra virus	Australia	I	Bat	[9]
Nipah virus Malaysia	Malaysia	I	Bat	[19]
Nipah virus Bangladesh	Bangladesh	I	Bat	[4]
Cedar virus	Australia	I	Bat	[31]
Mòjiāng virus	China	S	Cave rat	[35]
Ghanian bat virus	Ghana	S	Bat	[33]
Angavokely henipavirus	Madagascar	S	Bat	[38]
Denwin virus	Belgium	S	Shrew	[39]
Gamak virus	Korea	I	Shrew	[36]
Daeryong virus	Korea	S	Shrew	[36]
Langya virus	China	I	Shrew	[37]
Melian virus	Guinea	S	Shrew	[39]
Jingmen Crocidura shantungensis henipavirus 1	China	S	Shrew	OM030314.1
Jingmen Crocidura shantungensis henipavirus 2	China	S	Shrew	OM030315.1
Wufeng Chodsigoa smithii henipavirus 1	China	S	Shrew	OM030316.1
Wufeng Crocidura attenuata henipavirus 1	China	S	Shrew	OM030317.1
Wenzhou Apodemus agrarius henipavirus 1	China	S	Striped field mouse	MZ328275.1
Peixe-Boi virus	Brazil	S	Opossum	[40]
